# Selecting Native Arbuscular Mycorrhizal Fungi to Promote Cassava Growth and Increase Yield under Field Conditions

**DOI:** 10.3389/fmicb.2016.02063

**Published:** 2016-12-22

**Authors:** D. Jean-Marc Séry, Z. G. Claude Kouadjo, B. R. Rodrigue Voko, Adolphe Zézé

**Affiliations:** ^1^Laboratoire de Biotechnologies Végétale et Microbienne, Unité Mixte de Recherche et d'Innovation en Sciences Agronomiques et Génie Rural, Institut National Polytechnique Felix Houphouët-BoignyYamoussoukro, Côte d'Ivoire; ^2^Laboratoire Central de Biotechnologies, Centre National de la Recherche AgronomiqueAbidjan, Côte d'Ivoire; ^3^Unité de Formation et de Recherche en Agroforesterie, Université Jean Lorougnon GuédéDaloa, Côte d'Ivoire

**Keywords:** AMF, ecological engineering, cassava yield, tolerance, *Meloidogyne*, drought

## Abstract

The use of arbuscular mycorrhizal fungal (AMF) inoculation in sustainable agriculture is now widespread worldwide. Although the use of inoculants consisting of native AMF is highly recommended as an alternative to commercial ones, there is no strategy to allow the selection of efficient fungal species from natural communities. The objective of this study was (i) to select efficient native AMF species (ii) evaluate their impact on nematode and water stresses, and (iii) evaluate their impact on cassava yield, an important food security crop in tropical and subtropical regions. Firstly, native AMF communities associated with cassava rhizospheres in fields were collected from different areas and 7 AMF species were selected, based upon their ubiquity and abundance. Using these criteria, two morphotypes (LBVM01 and LBVM02) out of the seven AMF species selected were persistently dominant when cassava was used as a trap plant. LBVM01 and LBVM02 were identified as *Acaulospora colombiana* (most abundant) and *Ambispora appendicula*, respectively, after phylogenetic analyses of LSU-ITS-SSU PCR amplified products. Secondly, the potential of these two native AMF species to promote growth and enhance tolerance to root-knot nematode and water stresses of cassava (Yavo variety) was evaluated using single and dual inoculation in greenhouse conditions. Of the two AMF species, it was shown that *A. colombiana* significantly improved the growth of the cassava and enhanced tolerance to water stress. However, both *A. colombiana* and *A. appendicula* conferred bioprotective effects to cassava plants against the nematode *Meloidogyne* spp., ranging from resistance (suppression or reduction of the nematode reproduction) or tolerance (low or no suppression in cassava growth). Thirdly, the potential of these selected native AMF to improve cassava growth and yield was evaluated under field conditions, compared to a commercial inoculant. In these conditions, the *A. colombiana* single inoculation and the dual inoculation significantly improved cassava yield compared to the commercial inoculant. This is the first report on native AMF species exhibiting multiple benefits for cassava crop productivity, namely improved plant growth and yield, water stress tolerance and nematode resistance.

## Introduction

Cassava (*Manihot esculenta* Crantz) is a Central and South American native plant with tuberous roots rich in starch. It is a staple food for over 800 million people living in developing countries (Burns et al., 2010). In Côte d'Ivoire, it is the second most important food crop with an estimated annual production in 2013 of 2.5 million tons (FAO, 2014). This crop plays an important role in food security and income generation for many smallholder families. Despite its importance, cassava productivity is low in Côte d'Ivoire where yields are around 6 to 8t/ha compared to a global average level of 13t/ha (FAO, 2014). This low yield appears to be due to several factors. Firstly cassava cropping systems in Côte d'Ivoire are intensive and result in a rapid loss of soil fertility usually requiring long fallow periods (up to 7 years) to restore phosphorus and nitrogen levels. Secondly, cassava productivity is affected by pests, with root-knot nematodes being of major importance (Caveness, 1982; McSorley et al., 1983; Jatala and Bridge, 1990). Cassava yield losses due to nematode damage can be up to 87% (Caveness, 1982; IITA, 1990). Root-knot nematode damage can now be considered as a threat to the production of this major crop plant in Côte d'Ivoire where producers are mainly small farmers who cannot afford to buy nematicides. A third problem is the effect of climate change on crop productivity; notably the impact of drought, as unreliability of rainfall is a factor limiting cassava cultivation in tropical and subtropical areas (N'Guettia and Bernard, 1986). Consequently, although known for its ability to withstand drought, the net biomass production of cassava is reduced in times of water stress, irrespective of variety (Connor et al., 1981). Therefore, in order to sustain cassava productivity for farmers in tropical regions, it is important to develop a technology that can confer simultaneously on this plant (i) better growth and yield, (ii) a better tolerance to nematodes and (iii) a better tolerance to water deficit. Compounding the effects of disease and water stress is the increased vulnerability of rural families and smallholder cassava producers who often do not have access to appropriate technologies, services and markets. In developed countries, farmers rely extensively on industrial fertilizers to maximize crop productivity. Unfortunately, because of their financial and environmental costs, industrial fertilizers are not a solution for underdeveloped countries (Sanchez, 2002).

Cassava farmers could benefit from the multiple services offered by soil microorganisms such as arbuscular mycorrhizal fungi (AMF). Indeed, AMF belonging to the phylum Glomeromycota (Schüßler et al., 2001), constitute a multifunctional partner in the mutualistic interaction they develop with most land plants. The major function of AMF is to provide the mycorrhizal plant with water and essential nutrients such as phosphorus and nitrogen (He et al., 2003; Smith and Read, 2008). In addition to this nutritional function they provide, AMF can enhance plant tolerance to both biotic and abiotic stresses (Augé, 2001, 2004; Ortas et al., 2001; Plenchette et al., 2005; Al-karaki, 2006; Pozo and Azcón-Aguilar, 2007; Porcel et al., 2011; Augé et al., 2015). This multifunctional ability of the partner fungi has led to the development of mycorrhizal inoculants as biofertilizers in agriculture. Mycorrhizal inoculation has been applied for decades to promote better plant growth for various crop plants (Osonubi et al., 1995; Carretero et al., 2009). Cassava is highly mycorrhizal (Sieverding, 1989; Oyetunji and Osonubi, 2007) and there is evidence that AMF play an important role in increasing the productivity of cassava (Sieverding, 1989; Cardoso and Kuyper, 2006; Ceballos et al., 2013). Despite this positive impact of AMF inoculation on cassava productivity, and the known positive impact of mycorrhizal inoculation on root-knot nematode infection in crops such as yam and grapevine (St-Arnaud and Vujanovic, 2007; Tchabi, 2008; Hao et al., 2012; Veresoglou and Rillig, 2012), studies of AMF effects on root-knot nematode and water deficit in cassava remain scarce. In other studies, the impact of AMF on water stress has been documented for several crop plants, including cassava (Augé, 2001; Oyetunji et al., 2007). Although these studies point out the importance of AMF, there is no report of an AMF species that can (i) promote cassava growth and yield (ii) alleviate root-knot nematode damage and (iii) alleviate water stress. Therefore, the identification of AMF exhibiting these three traits could be a step forward to sustain cassava productivity in tropical regions.

The objective of this work was to recover native AMF species from smallholder farms and evaluate their potential to promote cassava growth and enhance resistance to root-knot nematode and water stress. Criteria such as ubiquity and relative abundance in field soils and baited soils were used to select native AMF species that were subsequently further evaluated for plant growth promotion in greenhouse and field conditions.

## Materials and methods

### Plant material

The improved cassava variety TME 7 “Yavo” provided by the National Agency for Rural Development Support (ANADER) in Yamoussoukro was used for the experiment. This variety has an 8-month cycle and is known to be resistant to the African cassava mosaic virus. In general, cassava leaves and roots are well developed after 4 months. At this stage, biotic and abiotic stresses can affect cassava growth parameters (Connor et al., 1981). Therefore all the experiments in greenhouse were run on 4-month-old cassava plants.

### Methods

#### Selecting potential arbuscular mycorrhizal fungi for inoculum development

##### Recovery of AMF species from field soils

Soil samples were collected from cassava fields during the dry period in December 2012 in three agro-ecological zones (Azaguié, Yamoussoukro and Abengourou), which are considered important cassava production areas in Côte d'Ivoire (Chaleard, 1988; Kouadio et al., 2010). Twelve soil samples (1 kg each) were collected at a depth of 0–20 cm from cassava plant rhizospheres, using the sampling method of Huang and Cares (2004), from four fields in each agro-ecological zone (Table 1). AMF were extracted from 50 g of field soils by wet sieving (Gerdemann and Nicolson, 1963) using 4 sieves (45, 90, 125, and 500 μm). AMF species were identified as described below, and selected according to abundance, occurrence and ubiquity. Species occurrence was determined as the number of fields in which a particular species was found divided by the total number of fields. Each morphotype was maintained in monoculture using variety “Yavo” as a host, in 2-L pots containing soil + sand (3:1 v/v) sterilized by autoclaving.

**Table 1:** **Geographic coordinates of fields**.

**Zone**	**Field**	**Area (ha)**	**Point**	**Geographic coordinates**		
				**North**	**West**	**Altitude (m)**
ABENGOUROU	Aniansué 1 (AB1)	1–2	Ab 1/1	06°40′20.10″	003°38′57.72″	166
			Ab 1/2	06°40′20.64″	003°38′56.34″	164
			Ab 1/3	06°40′20.28″	003°38′58.56″	164
	Aniansué 2 (AB2)	2–3	Ab 2/1	06°39′51.96″	003°41′07.80″	170
			Ab 2/2	06°39′53.82″	003°41′06.66″	167
			Ab 2/3	06°39′50.76″	003°41′06.06″	164
	Dramanekro 1 (AB3)	1–2	Ab 3/1	06°42′38.40″	003°37′03.36″	176
			Ab 3/2	06°42′37.44″	003°37′04.80″	176
			Ab 3/3	06°42′37.32″	003°37′05.34″	177
	Dramanekro 2 (AB4)	1–2	Ab 4/1	06°41′48.96″	003°38′19.08″	151
			Ab 4/2	06°41′50.82″	003°38′17.94″	154
			Ab 4/3	06°41′51.60″	003°38′16.50″	152
AZAGUIE	Ahoua 1 (AZ1)	1–2	Az 1/1	05°40′21.06″	004°02′33.42″	51
			Az 1/2	05°40′22.38″	004°02′32.64″	50
			Az 1/3	05°40′22.86″	004°02′31.38″	50
	Ahoua 2 (AZ2)	1–2	Az 2/1	05°38′36.30″	004°03′24.54″	48
			Az 2/2	05°38′34.32″	004°03′18.36″	47
			Az 2/3	05°38′35.88″	004°03′21.36″	46
	M'Bromé 1 (AZ3)	2–3	Az 3/1	05°39′38.28″	004°09′00.00″	53
			Az 3/2	05°39′37.14″	004°08′57.60″	49
			Az 3/3	05°39′35.94″	004°08′57.54″	47
	M'Bromé 2 (AZ4)	2–3	Az 4/1	05°40′04.98″	004°08′43.44″	46
			Az 4/2	05°40′01.56″	004°08′43.32″	47
			Az 4/3	05°39′58.74″	004°08′43.32″	52
YAMOUSSOUKRO	Logbakro 1 (YA1)	1–2	Ya 1/1	06°44′13.50″	005°12′24.60′'	223
			Ya 1/2	06°44′14.28″	005°12′24.60″	225
			Ya 1/3	06°44′15.42″	005°12′23.10″	225
	Logbakro 2 (YA2)	1–2	Ya 2/1	06°44′01.68″	005°11′44.22″	207
			Ya 2/2	06°44′02.64″	005°11′45.60″	210
			Ya 2/3	06°44′02.34″	005°11′46.86″	210
	Céman (YA3)	1–2	Ya 3/1	06°53′14.46″	005°17′54.96″	237
			Ya 3/2	06°53′15.06″	005°17′54.90″	233
			Ya 3/3	06°53′15.42″	005°17′54.00″	235
	Zambakro (YA4)	2–3	Ya 4/1	06°43′30.12″	005°24′15.48″	162
			Ya 4/2	06°43′30.36″	005°24′14.52″	159
			Ya 4/3	06°43′28.68″	005°24′14.16″	159

##### Isolation of abundant AMF species by trapping

Field soils were used to trap AMF species using the cassava variety “Yavo.” The collected soils were mixed with a substrate composed of a mixture of soil and sand (3:1,v/v) sterilized at 120°C and 2 bars for 1 h on two successive days (Bâ et al., 2000) in a 1:1 ratio (v/v). Soils were placed in 10-L pots. The pots were watered every other day with 400 ml of water without fertilizer. After 4 months, cassava plants showed good physiological development. Soils were carefully recovered using a spatula after 4 months of cultivation. 50 g of soils were used to identify abundant and ubiquitous AMF morphotypes.

#### Morphological identification of selected AMF spores

Spores were extracted by wet sieving and mounted between slide and coverslip in polyvinyl-lacto-glycerol and Melzer's solution (Morton et al., 1993). They were observed under a microscope and morphologically identified based on their color, shape, and composition of their walls (Schenck and Perez, 1990; see http://invam.wvu.edu/, http://www.zor.zut.edu.pl/ collection websites). Spores were photographed using a Motic BA310 Trinocular compound microscope.

#### Molecular identification of selected AMF morphotypes

In order to confirm the morphological identification of the selected AMF species, PCR amplification was performed using primers LR1-LSUmAr/LR1- LSUmBr. For each selected species, 10 spores were collected in a 1.5 ml microfuge tube for DNA extraction using the DNeasy Plant Mini Kit (Qiagen). A first PCR amplification using primers LR1 and LSUmAr (van Tuinen et al., 1998; Stockinger et al., 2009) and a nested PCR using LR1 and LSUmBr primers (Krüger et al., 2009; Stockinger et al., 2010) were performed in 30 cycles (95°C 5 min; 94°C 1 min; 58°C 30 s; 72°C 45 s; 72°C 5 min; 25°C 1 s). The size of PCR products were checked on 1% agarose gels. For sequencing, the amplified PCR products were purified using a commercial kit (Nucleospin Extract II) and cloned using the TOPO TA Cloning® Kit (Invitrogen) according to the manufacturer's instructions. Three positive clones were selected for sequencing by GATC Biotech (Konstanz, Germany) using the directional Sanger method. Sequence analyses were done by Blast with NCBI and MAARJAM databases and phylogenetic analyses were performed using the software MEGA 6.06 and the neighbor-joining method (Saitou and Nei, 1987).

#### Mycorrhizal inoculum production

To produce inoculum, the selected strains were grown individually in the greenhouse in a sterile substrate containing cassava plants. For single inoculation, the inoculum (S1: *A*. *colombiana* or S2: *A*. *appendicula*) was in the form of 50 g of sterile substrate (soil + sand; 3:1, v/v) containing pieces of mycorrhizal roots, hyphae and about 350 AMF spores. The soil characteristics were (pH = 7.1; organic matter = 2.81%; total nitrogen = 0.15%; available phosphorus = 55 mg/kg) and for the sand (pH = 6.7; organic matter = 0.17%; total nitrogen = 0.01%; available phosphorus = 2 mg/kg). For dual inoculation, the two inocula (25 g each) were mixed to make 50 g.

#### Evaluation of the impact of selected AMF on cassava growth and phosphorus status in greenhouse

##### Experimental design and culture condition

The greenhouse experiment was conducted comparing three AMF combinations (S1, S2, and S1S2) plus the control S0, and 6 replicates (completely randomized blocks) over 4 months. Pots were filled with 8 kg of substrate (soil + sand; 3:1, v/v). Each pot contained one cassava plant that was watered every other day with 400 ml of water without fertilizer.

##### Assessment of mycorrhizal development

For assessment of root colonization by AMF, fine cassava roots were sampled 4 months after planting, with three replicates per treatment. Each treatment contained three plants. Roots were rinsed and cut into 1–2 cm fragments. These roots fragments were cleared by boiling in 10% (w/v) KOH and stained with 0.05% (v/v) trypan blue in lactoglycerol according to the method of Phillips and Haymann (1970). Ten pieces of roots per plant were placed in glycerol (50%) between slide and coverslip (Kormanik and McGraw, 1982) and observed under an optical microscope. The colonized roots were observed and evaluated according to Trouvelot et al. (1986).

#### Assessment of the mycorrhizal inoculation on cassava growth and phosphorus levels in greenhouse

Plant growth was assessed by measuring plant height and foliar surface area using Connor's et al. methods (Connor et al., 1981), and total fresh and dry matter. Plant total fresh matter was determined using an OHAUS balance and the dry matter after oven drying at 80°C for 48 h. Eight young cassava leaves were analyzed for P content after 4 months by the mineralization and calcination method using a Tecator model 40 instrument (Sidney, 1984). All measurements were done in triplicate.

#### Evaluation of the impact of selected AMF species on cassava tolerance and resistance to root-knot nematode *Meloidogyne* spp. in greenhouse

##### Preparation of nematode inoculum

The nematode inoculum was made using a population of *Meloidogyne* spp., isolated from tomato galls grown in a greenhouse. The inoculum was prepared by finely cutting infected tomato roots that were soaked in a jar containing NaClO (0.25%) and shaken for 2 min (Hussey and Barker, 1973). Nematode eggs and juveniles were collected on a 25 μm sieve, rinsed in sterile water and counted under a 40x binocular magnifier. A suspension of 1000 nematodes (juveniles + eggs) was added to each cassava plant (each pot).

##### Experimental design and culture condition

A 4 × 2 factorial experiment with three replicates and completely randomized design was carried out in the greenhouse over a 4-month period. One factor was the AMF treatment: each selected AMF strain was used either in single inoculation (S1, S2) or dual inoculation (S1S2) and a non-inoculated control (S0). The other factor was inoculation with root knot nematodes, either at the same time as the AMF inoculation (I2), or 1 month after AMF inoculation (I4). Inoculation with nematodes was achieved by loading aliquots of 1000 freshly hatched juveniles and eggs suspended in distilled water into 5-cm-deep holes equidistant around each plant. Pots were filled with 8 kg of substrate (soil + sand; 3:1, v/v). Each pot contained one cassava plant that was watered every other day with 400 ml of water without fertilizer.

##### Assessment of the mycorrhizal inoculation impact on nematode population

At the end of the experiment, nematodes and eggs were counted according to Daykin and Hussey (1985). The total content of phenols, which are an indicator of plant defense compounds against nematode attack, in roots was estimated using a colorimetric method (Singleton et al., 1999). Total phenol content was measured after 2 h at room temperature incubation by absorbance at 765 nm, measured in a Jenway 7315 Spectrophotometer. The quantification was done using a gallic acid calibration curve. Biomass (total fresh matter, total dry matter) and phosphorus in cassava leaves were also determined as described above.

#### Impact of the selected AMF inoculation on cassava resistance to water stress in greenhouse

##### Experimental design and culture condition

Before the main experiment, a pot containing 8 kg of soil was filled with water until saturation. Excess water was then allowed to drain over 2 days and field capacity (FC) was measured according to Colombani et al. (1973). A 4 × 2 factorial experiment with three replicates and a completely randomized design was carried out in a greenhouse over a 4-month period. One factor was the AMF treatment: each selected AMF strain applied either in single inoculation (S1, S2) or dual inoculation (S1S2), and an non-inoculated control (S0). The other factor was water regime. All plants were watered to 100% of FC for 2 months after planting. They were then divided in two groups for the remaining 2 months. One group was regularly watered to 100% FC while the other was watered to 10% of FC. That watering regime corresponded to 400 mm of water/year, which can be considered a severe water stress to cassava (FAO, 2013). The total number of plants for the experiment was 48. Mycorrhizal abundance was estimated on roots harvested monthly using the Trouvelot et al. (1986) method. Foliar surface areas were measured on the 3rd and 4th month, and biomass was determined after 4 months, as described previously. The chlorophyll a content of young plant leaves was determined using the method of Arnon (1949). Soluble sugar content (TS) of young leaves, a measure of osmoprotection during water stress, was determined according to Dubois et al. (1956) using the Jenway 7315 spectrophotometer.

#### Cassava plant inoculation under field conditions

##### Study area

An experimental area of about 2500 m^2^ was set up in Duokro, 15 km from Yamoussoukro in Côte d'Ivoire, to test the effect of local and commercial strains of mycorrhizae on colonization, and cassava yield during the 2015–2016 season. The average temperature in this region over the season was 32 ± 2°C, average total annual rainfall is 1495 mm and average annual humidity is 79 ± 12%.

##### Experimental design

The field experiment was established using a randomized complete block design with five inoculation treatments: *Ambispora appendicula* (T1), *Acaulospora colombiana* (T2), the dual inoculant *A. colombiana–A. appendicula* (T3), a commercial inoculum Mykepro P501 produced by PremierTech biotechnologies (T4), and a non-inoculated control (T5). The commercial inoculant is composed of a single species *Rhizophagus intraradices*. Mineral fertilizer (30 kg N/ha, 20 kg P/ha and 50 kg K/ha) was applied to the non-inoculated control plots. For each treatment, there were three replicates, resulting in a total of 18 plots. Cassava was planted in ridges 80 cm wide and 20 cm high, separated by 20-cm wide furrows, following the contour. The blocks were arranged perpendicular to the slope. Each plot contained 40 plants, including 16 inoculated plants and 2 lines of 24 plants curbs to limit edge effects. The planting density was 10,000 plants/ha. 25-cm long cassava cuttings, 1.5–2.5 cm thick, with 5–6 nodes were planted in 20-cm deep holes. Cuttings were inserted diagonally in order to promote sprouting. No irrigation water or pesticides were applied. Cassava plant inoculation was done on farm. For the native inoculum, each plant was inoculated with 100 g of inoculums containing 1000 spores + mycorrhizal roots. For the commercial inoculum 6 g containing 3000 spores + mycorrhizal roots was added to each plant, corresponding to triple the dose applied in temperate zones.

##### Assessment of cassava tuber yield

Tubers were harvested on March 10, 2016. Fresh tubers were weighed and the yield converted to t/ha. When a significant difference was observed in yield compared to plots without AMF, the gain (G) in yield was calculated according to the formula:

$$G\left(\%\right)=100\;*\;\left(\frac{\text{Yield with AMF}-\text{Yield without AMF }\left(\text{control}\right)}{\text{Yield without AMF }\left(\text{control}\right)}\right)$$

#### Statistical analyses

All experimental data in greenhouse were subjected to statistical analyses by performing either one or two-way analysis of variance (ANOVA) using Statistica 7.1. The significance of the treatment effects was determined using LSD Fisher test with *P* = 0.05.

All field experiment data were analyzed by ANOVA. Fisher's LSD test was also used to determine whether or not treatments were different from each other at *P* < 0.05.

## Results

### Selection and identification of potential useful AMF species for cassava crop inoculation

**Figure 1:**
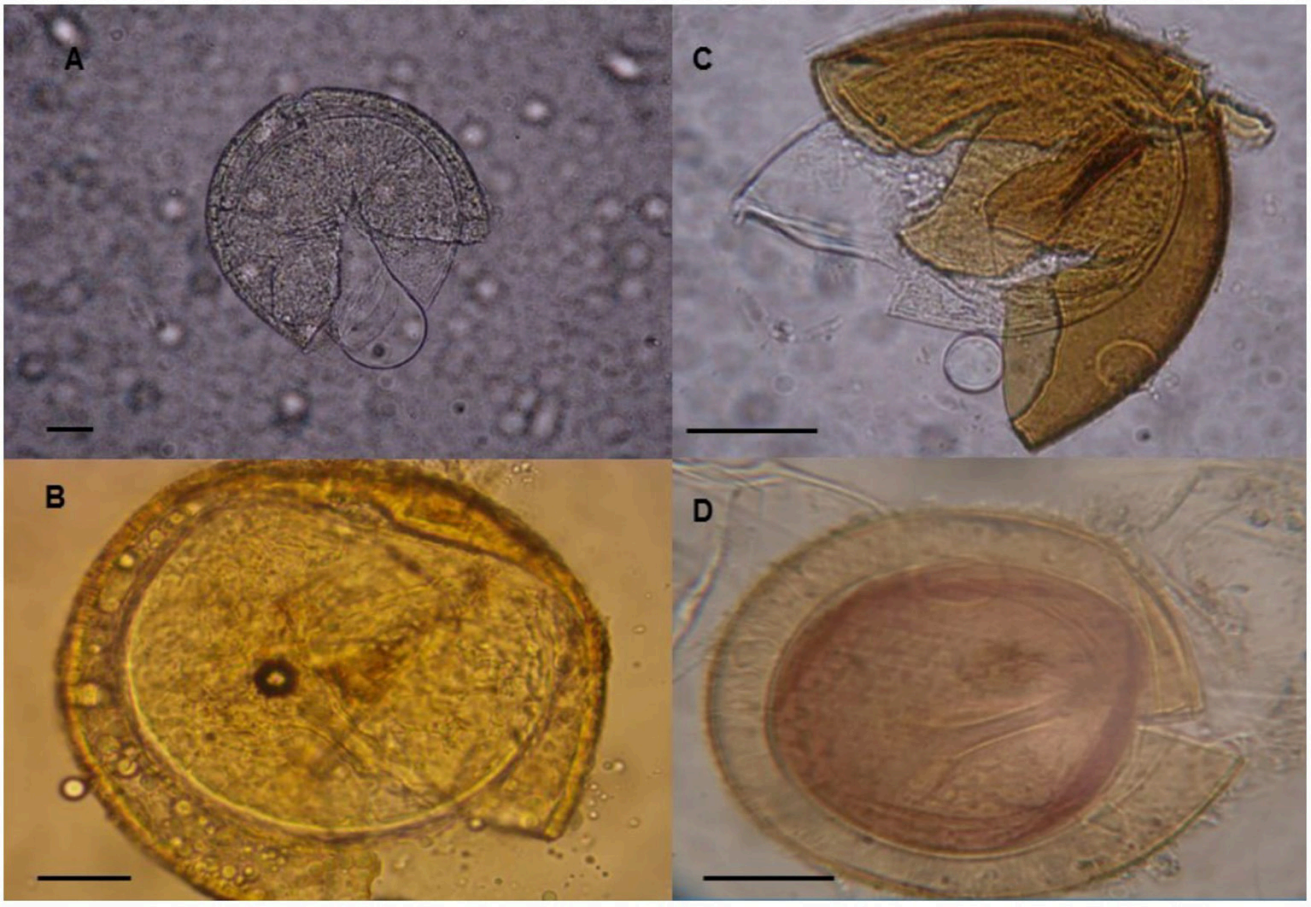
**Spores of arbuscular mycorrhizal fungi used in this study. (A)** *Acaulospora colombiana* and **(C)** *Ambispora appendicula* stained with lactoglycerol polyvinyl. **(B)** *Acaulospora colombiana* and **(D)** *Ambispora appendicula* stained with Melzer's reagent. Scale bar = 1/25 μm.

**Figure 2:**
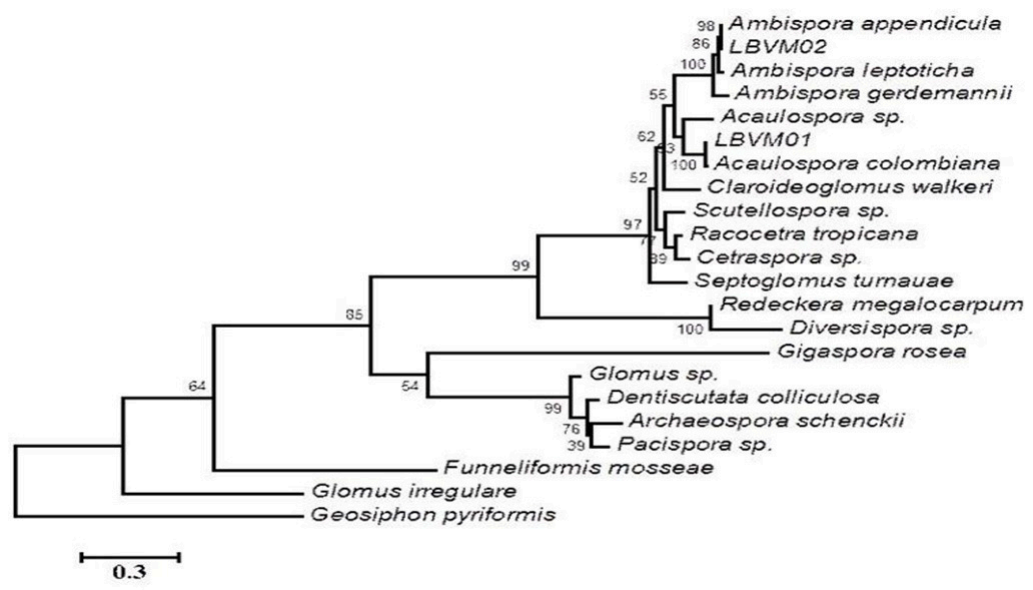
**Phylogenetic identification of LBVOl and LBVM02 isolated from field soils**. SSU-ITS-LSU gene sequences from AMFs species *Ambispora appendicula* (FN547527), *Ambispora leptoticha* (KC166277), *Ambispora gerdemanii* (KC166282), *Acaulospora* sp. (HF56794), *Acaulospora colombiana* (FR750063), *Scutellospora* sp. (AF396818), *Racocetra tropicana* (GU385898), *Cetraspora* sp. (HM565946), *Septoglomus tumauae* (KF060327), *Redeckera megalocarpum* (NR121478), *Diversispora* sp. (KJ850185), *Gigaspora rosea* (U60451), Glomus sp. (AB326023), *Dentiscutata colliculosa* (GQ376067), *Archaeospora schenckii* (KP144303), *Claroideoglomus walker* (KP191492); *Pacispora* sp. (JQ182768), *Funneliforrnis mossae* (KM360085), *Glomus irregulare* (GU585513) and *Geosiphon pyriformis* (JX535577) were used for comparison. The tree was constructed by the neighbor-joining method using Mega version 6.

Using spore characteristics, several AMF species were identified (Table 2) in the three agricultural zones. The species *Acaulospora scrobiculata, A. colombiana, A. appendicula, Claroideoglomus etunicatum, Glomus glomerulatum* and an unidentified species *Glomus Sp*2 were abundant at various levels in all three zones. However, when the cassava cultivar Yavo was inoculated with field soils in greenhouse, only *A. colombiana, A. appendicula* were confirmed in all soils, with *A*. *appendicula* having low abundance (Table 2). These two morphotypes (LBVM01 and LBVM02), which were present in all soils and also abundant in trapped communities, were considered as good candidates for cassava inoculation. They were initially identified based on morphological criteria using PVLG and Melzer's reagent as *Acaulospora* sp. and *Ambispora* sp. (Figure 1). An expected 700 bp fragment was amplified from each morphotype. Both BLAST and phylogenetic analyses allowed the identification of the morphotype LBVM01 as *A. colombiana* (Genbank accession number KX168435) and the other LBVM02 as *A. appendicula* (Genbank accession number KX168436) (Table 3; Figure 2).

**Table 2:** **Abundance of efficient arbuscular mycorrhizal fungi (AMF) species**.

**AMF species**	**AB1**	**AB2**	**AB3**	**AB4**	**AZ1**	**AZ2**	**AZ3**	**AZ4**	**YA1**	**YA2**	**YA3**	**YA4**	**Occurrence (%)**
**NATIVE AMF SPECIES RECOVERED FROM CASSAVA FIELD SOILS**													
*Acaulospora excavata*	+	+	+	+	–	–	–	–	++	+	+	+	67
*Acaulospora scrobiculata*	++	+++	+++	++	++	+	++	+	++	++	++	++	100
***Acaulospora columbiana***	**+**	**+**	**++**	**++**	**+++**	**++**	**+**	**++**	**++**	**+**	**++**	**++**	**100**
***Ambispora apendicula***	**+++**	**++**	**++**	**+++**	**+++**	**+++**	**+++**	**+++**	**+**	**++**	**+**	**++**	**100**
*Claroideoglomus etunicatum*	++	++	++	++	+	+	+	+	++	++	++	++	100
*Glomus aureum*	–	+	+	+	+	+	+	+	–	–	–	–	58
*Glomus glomerulatum*	++	+	++	++	++	++	++	++	+++	+++	+++	++	100
*Glomus clavisporum*	–	+	+	–	–	++	++	–	–	–	–	++	42
*Glomus* sp.1	++	+	++	+	–	–	–	–	+	++	++	++	67
*Glomus* sp.2	++	+++	++	++	+++	++	+++	+++	+++	++	++	++	100
*Funneliformis mossae*	–	–	–	–	+	–	–	–	–	–	+	–	17
*Rhizophagus intraradices*	++	++	++	+++	–	–	–	–	+	+	+	+	67
*Rhizophagus manhiotis*	–	–	–	–	+	+	–	–	–	–	–	–	17
*Sclerocystis sinuosum*	+	–	–	++	+	+	++	–	+	–	+	+	67
*Septoglomus constrictum*	–	–	–	–	+	++	+	–	–	–	+	–	33
*Gigaspora*sp.1	–	–	–	–	–	+	–	–	–	–	–	–	8
*Racocetra africana*	–	–	–	–	+	+	+	+	+	+	+	+	67
*Scutelospora* sp.	+	–	+	+	+	+	+	+	–	+	+	+	83
**AMF SPECIES TRAPPED AFTER 4 MONTHS FROM FIELD SOILS USING THE CASSAVA CULTIVAR YAVO**													
*Acaulospora scrobiculata*	++	+	+++	–	–	–	+	+++	+	–	+++	–	58
***Acaulospora columbiana***	**+++**	**+++**	**+++**	**+++**	**+++**	**+++**	**+++**	**+++**	**++**	**+++**	**+++**	**++**	**100**
*Acaulospora*sp.1	+	+	–	–	+	+	–	–	–	–	–	–	33
***Ambispora appendicula***	**+**	**+**	**+**	**+**	**+**	**+**	**+**	**+**	**+**	**+**	**+**	**+**	**100**
*Glomus clavisporum*	++	++	–	–	–	+++	++	++	+	–	–	+	58
*Rhizophagus intraradices*	++	++	++	+++	–	–	–	–	+	+	+	+	67
*Gigaspora* sp.1	++	++	+	+	++	+	–	+	–	–	–	–	58

*AB, Abengourou; AZ, Azaguié; YA, Yamoussoukro; Field number, 1–2–3–4. –, absent (0 spore/g); +, present (1–2 spores/g); ++, abundant (3–5 spores/g); +++, highly abundant (6–8 spores/g). bold indicates abundant and ubiquitous AMF species in both field soils and trapped culture*.

**Table 3:** **Consensus identification of the two native species of arbuscular mycorrhizal fungi**.

**Morpho-species**	**Morphological identification**	**Molecular identification**	**Consensus species**	**Species accession number in databases**
S1	*Acaulospor*a sp.	*A. colombiana*	*A. colombiana*	KX168435
S2	*Ambispora* sp.	*A. appendicula*	*A. appendicula*	KX168436

### Effect of *A. colombiana* and *A. appendicula* single and dual inoculation on cassava growth and P uptake

After 4 months of culture in greenhouse conditions (Table 4), all cassava plants inoculated (singly or dually) with *A. colombiana* and *A. appendicula* were mycorrhizal. Frequencies and intensities of mycorrhization did not differ significantly between single and dual inoculation (frequency of 26.7 and 48.3%, and intensity of 14.5 and 38.7%, respectively). No mycorrhizal structures were observed in cassava control plants. The foliar P content of cassava plants inoculated with *A. colombiana* was significantly (*p* = 0.002) improved (1.3-fold) compared to the non-inoculated control, whereas the *A. appendicula* single inoculation had no impact. The foliar P content of dual inoculated plants was significantly improved (1.5-fold) compared to the control. The growth parameters (plant height, foliar surface area, total dry and total fresh matter) of *A. colombiana* singly inoculated cassava plants were significantly improved (*p* = 0.040; *p* = 0.008; *p* = 0.000; *p* = 0.001, respectively) after 4 months in the greenhouse. The *A. appendicula* single inoculation had significant impact only on total fresh matter. However, dual inoculation significantly improved all parameters.

**Table 4:** **Impact of ***A. colombiana*** and ***A. appendicula*** single and dual inoculation on phosphorus nutrition and cassava growth after 4 months**.

**Treatment**	**Frequency of mycorrhization (%)**	**Intensity of mycorrhization (%)**	**Plant height (cm)**	**Foliar surface area (cm^2^)**	**Total fresh matter (g)**	**Total dry matter (g)**	***P* (%dm)**
Control (S0)	0 ± 0	0 ± 0	34.1^c^ ± 0.9	898.3^c^ ± 38.96	47.4^c^ ± 2.5	15.9^c^ ± 0.2	0.17^c^ ± 0.008
*A. colombiana* (S1)	48.3^a^ ± 11.8	25.4^a^ ± 7.5	41.5^ab^ ± 0.7	1375.6^ab^ ± 162.3	56.9^ab^ ± 3.2	18.3^b^ ± 0.7	0.22^b^ ± 0.0
*A. appendicula* (S2)	26.7^a^ ± 4.5	14.5^a^ ± 3	36.7^bc^ ± 2.1	1219.1^bc^ ± 80.36	55^b^ ± 1	17.4^bc^ ± 0.6	0.19^bc^ ± 0.0
*A. colombiana-A. appendicula (S1S2)*	46.7^a^ ± 15.6	38.7^a^ ± 12.8	44.33^a^ ± 2.6	1711.6^a^ ± 54.01	60.7^a^ ± 2.9	20.3^a^ ± 2.2	0.26^a^ ± 0.016
***P* and *F*-VALUE**							
	*p = 0.018*	*p = 0.024*	*p = 0.040*	*p = 0.008*	*p = 0.000*	*p = 0.001*	*p = 0.002*
	*F* = 4.19	*F* = 3.91	*F* = 4.454	*F* = 8.1851	*F* = 14.58	*F* = 7.93	*F* = 12.267

*dm, dry matter. All the values are means of the three replications (n = 3). Means with different letters were significantly different at 5% level*.

### Susceptibility of *A. colombiana* and *A. appendicula* inoculated cassava plants to root-knot nematode *Meloidogyne* spp

Four months after single or dual inoculation with *A. colombiana* or *A. appendicula*, in the presence of the nematode *Meloidogyne* spp., all treated cassava plants were mycorrhizal (Table 5). However, the frequencies and intensities of mycorrhization were significantly lower (*p* = 0.002 for both) in the roots of cassava plants that were co-inoculated with the nematode *Meloidogyne* spp. In this condition, the presence of *A. colombiana* and *A. appendicula* as single or dual inoculant significantly reduced nematode egg and population densities. In this experiment, none of the three mycorrhizal inoculation methods significantly affected foliar P contents. However, the single inoculation using *A. appendicula* and the dual inoculation significantly increased cassava total fresh biomass. When the nematodes were added 1 month after AMF inoculation, mycorrhizal root colonization levels were still high after 4 months (frequency 20 and 36.7%, and intensity 15 and 21.5% for single and dual inoculation, respectively). In this case, only the single inoculation with *A. colombiana* significantly (*p* = 0.006) reduced the number of nematode eggs in the cassava roots. Also, only *A. colombiana* as a sole inoculants significantly increased the foliar P content. However, only the dual inoculation increased cassava plant fresh and dry biomass. Phenol contents of AMF pre-inoculated cassava roots were significantly higher than the controls (not inoculated with AMF).

**Table 5:** **Impact of ***A. colombiana*** and ***A. appendicula*** single and dual inoculation on nematode communities and cassava plant growth**.

**Treatment**	**Frequency of mycorrhization (%)**	**Intensity of mycorrhization (%)**	**Egg density in the roots/g**	**Density of nematodes in the roots/g**	**Foliar P content**	**Biomass (g)**		**Phenols**
					**P (%dm.)**	**Total fresh matter (g)**	**Total dry matter (g)**	**Phenol concentration (mg EGA/l)**
**CO-INOCULATION WITH AMF AND NEMATODES (I2)**								
Control (S0)	0 ± 0	0 ± 0	3.7^c^ ± 0.47	7^b^ ± 4.24	0.21^ab^ ± 0.03	46^bc^ ± 1.5	14.1^ab^ ± 0.6	95^de^ ± 4.7
*Acaulospora colombiana* (S1)	6.7^b^ ± 4.7	0.07^b^ ± 0.04	2^ab^ ± 0.0	2^a^ ± 0.8	0.21^ab^ ± 0.01	53.7^abc^ ± 2.4	15.3^ab^ ± 0.2	116.7^cd^ ± 12
*Ambispora appendicula* (S2)	13.3^b^ ± 9.4	5.5^b^ ± 3.67	2^ab^ ± 0.81	2.33^a^ ± 1.24	0.22^abc^ ± 0.00	51.5^a^ ± 5.9	17.3^a^ ± 4.4	98.3^de^ ± 3.6
*A. colombiana—A. appendicula* (S1S2)	3.3^b^ ± 4.7	0.03^b^ ± 0.04	1.7^a^ ± 0.94	2^a^ ± 0.82	0.237^ab^ ± 0.0	56.3^a^ ± 7.2	15.6^a^ ± 2.26	115.7^cde^ ± 4.5
**INOCULATION WITH NEMATODES 1 MONTH AFTER AMF (I4)**								
Control (S0)	0 ± 0	0 ± 0	3.3^bc^ ± 1.24	5.17^ab^ ± 1.65	0.203^b^ ± 0.01	45.1^c^ ± 3.25	11.7^b^ ± 0.22	88.5^e^ ± 4
*Acaulospora colombiana* (S1)	30^a^ ± 8.16	15.07^a^ ± 3.2	1.7^a^ ± 0.47	2.17^a^ ± 0.13	0.255^a^ ± 0.00	53.5^abc^ ± 1.55	15.36^ab^ ± 0.05	169.5^a^ ± 4
*Ambispora appendicula* (S2)	20^a^ ± 8.16	15.07^a^ ± 6.3	2^ab^ ± 0	2.67^a^ ± 0.47	0.213^ab^ ± 0.00	54.34^ab^ ± 4.94	14.85^ab^ ± 1	126^bc^ ± 13.8
*A. colombiana—A. appendicula* (S1S2)	36.7^a^ ± 9.4	21.4^a^ ± 6.08	2^ab^ ± 0	2.17^a^ ± 0.13	0.227^ab^ ± 0.01	55.5^a^ ± 3.75	17.9^a^ ± 1.13	150.17^ab^ ± 4.24
***P* and *F*-VALUE**								
AMF	*p = 0.002*	*p* = 0.002	*p* = 0.006	*p* = 0.012	*p* = 0.47	*p* = 0.02	*p* = 0.045	*p* = 0.000
	*F* = 7.729	*F* = 7.8	*F* = 5.89	*F* = 5.044	*F* = 0.887	*F* = 4.35	*F* = 3.364	*F* = 12.52
Nematodes	*p = 0.000*	*p = 0.000*	*p = 0.806*	*p = 0.741*	*p = 0.97*	*p = 0.91*	*p = 0.5*	*p = 0.000*
	*F* = 22.5625	*F* = 41.77	*F* = 0.0625	*F* = 0.113	*F* = 0.001	*F* = 0.013	*F* = 0.47	*F* = 17.72
AMF × Nematodes	*p = 0.010*	*p = 0.004*	*p = 0.874*	*p = 0.785*	*p = 0.234*	*p = 0.92*	*p = 0.24*	*p = 0.033*
	*F* = 5.23	*F* = 6.483	*F* = 0.223	*F* = 0.357	*F* = 1.573	*F* = 0.17	*F* = 1.53	*F* = 3.7

*dm, dry matter, EGA, Equivalent Gallic Acid. All the values are means of the three replications (n = 3). Means with different letters were significantly different at 5% level*.

### Development of single and dual *A. colombiana* and *A. appendicula* inoculated plants during water stress

When cassava plants were well watered (100% FC), mycorrhizal colonization significantly increased during the first 2 months when inoculated with *A. colombiana* (from 23 to 46.7%) and with the dual inoculation (10 to 23.7%), but not with *A. appendicula* (constant at 10%) (Table 6). One month after water stress was initiated (10% FC), mycorrhizal colonization declined significantly in the cassava roots for all mycorrhizal treatments (Table 6). After 2 months of water stress the same trend was observed for all mycorrhizal treatments. However, *A. colombiana* colonization of cassava roots remained stable at 26.7% in the fourth month and this was significantly higher (*p* = 0.049) than *A. appendicula*. Under water stress, the presence of *A. colombiana* had a significant positive impact on the growth parameters (total dry matter, fresh matter and foliar surface area) (Table 7). In comparison, neither the single inoculation with *A. appendicula* nor the dual inoculation positively impacted these growth parameters. Moreover, under this severe water stress, the cassava plants inoculated with *A. colombiana* significantly improved all functional traits measured, including chlorophyll a (0.209 mg/g FM) and total sugar content (496 μmol/mg FM), compared to non-inoculated plants (Table 7).

**Table 6:** **Mycorrhizal frequency evolution from well-watered regime (100% of field capacity) at 1 to 2 months, to drought (10% of field capacity) at 2–4 months**.

**AMF frequency**	**1 month**	**2 months**	**3 months**	**4 months**
*Acaulospora colombiana*	23.3^a^ ± 7.2	46.7^a^ ± 11.86	23.3^a^ ± 4.714	26.7^a^ ± 4.7
*Ambispora appendicula*	10^a^ ± 0	10^b^ ± 0	20^a^ ± 2.72	10^b^ ± 4.7
*A. colombiana-A. appendicula*	10^a^ ± 0	23.3^ab^ ± 5.44	23.3^a^ ± 7.2	16.7^b^ ± 1.3
Control	0 ± 0	0 ± 0	0 ± 0	0 ± 0
***P*- and *F*-VALUE**				
	*p = 0.035*	*p = 0.016*	*p = 0.048*	*p = 0.006*
	*F* = 4.714	*F* = 6.377	*F* = 4.121	*F* = 9.067

*Means with different letters were significantly different at p < 0.05*.

**Table 7:** **Impact of ***A. colombiana*** and ***A. appendicula*** single and dual inoculation on cassava plant growth and physiological traits 2 months after initiation of water stress**.

**AMF**	**Foliar surface area (cm**^**2**^**)**		**Total fresh matter**		**Total dry matter**		**Chlorophyll a (mg/g FM)**		**Total sugar content (**μ**mol/mg FM)**	
	**Well-watered**	**Drought**	**Well-watered**	**Drought**	**Well-watered**	**Drought**	**Well-watered**	**Drought**	**Well-watered**	**Drought**
Control (S0)	4362.67^b^ ± 496	39.4^c^ ± 50.9	218.9^b^ ± 4.2	139^d^ ± 4.6	106.74^c^ ± 6.7	88.7^d^ ± 3.6	0.178^bc^ ± 0.0	0.123^cd^ ± 0.0	518^a^ ± 43.7	362.5^b^ ± 7
*A.colombiana (S1)*	6179.9^a^ ± 608.3	145.5^c^ ± 23.2	243.53^a^ ± 14.5	180^c^ ± 8.9	149.3^a^ ± 8.32	109.07^c^ ± 9.97	0.164^abc^ ± 0.0	0.209^a^ ± 0.0	523.4^a^ ± 23.5	496^a^ ± 32.4
*A.appendicula (S2)*	4872.3^b^ ± 589.3	13.3^c^ ± 17.17	231.35^ab^ ± 2.56	156^d^ ± 6.1	130.97^b^ ± 4.96	78.37^d^ ± 4.6	0.17^bc^ ± 0.01	0.078^d^ ± 0.0	480.4^ac^ ± 10.7	435.6^abc^ ± 26
*A.colombiana—A.appendicula (S1S2)*	4617.3^b^ ± 419.4	52.3^c^ ± 49.5	217.03^b^ ± 12.4	153^d^ ± 11.8	109.7^c^ ± 3.3	87.87^d^ ± 8.7	0.18^bc^ ± 0.01	0.15^bc^ ± 0.01	446.5^abc^ ± 29.8	351.5^bc^ ± 25
**P and F*****-*****VALUE**										
AMF	*p = 0.002*		*p = 0.000*		*p = 0.000*		*p = 0.017*		*p = 0.032*	
	*F* = 5.7652		*F* = 9.970		*F* = 19.240		*F* = 4.7855		F = 3.769	
Water regime	*p = 0.000*		*p = 0.000*		*p = 0.000*		*P = 0.010*		*p = 0.009*	
	*F* = 763.3963		*F* = 238.107		*F* = 97.697		*F* = 8.7654		F = 8.748	
Strain × Water regime	*p* = 0.008		*p* = 0.492		*P* = 0.007		*P* = 0.009		P = 0.449	
	*F* = 4.4874		*F* = 0.838		*F* = 5.815		*F* = 5.6544		F = 0.928	

*FM, Fresh matter. All the values are means of the three replications (n = 3). Means with different letters were significantly different at 5% level*.

### Impact of mycorrhizal inoculation on cassava yield under field conditions

**Figure 3:**
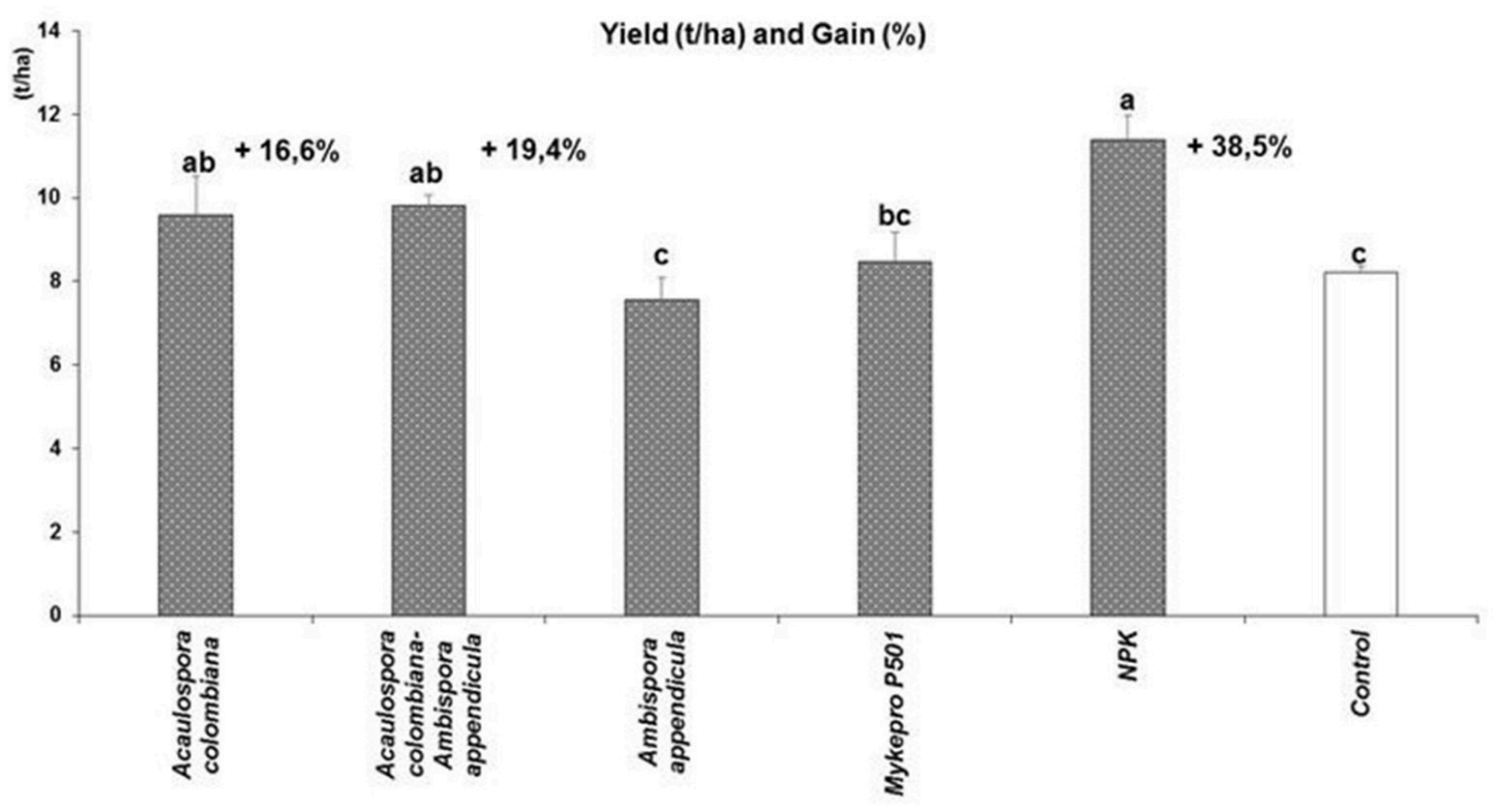
**Yield and yield gain of fresh cassava tubers as affected by inoculation with arbuscular mycorrhizal fungi or application of chemical fertilizer, under field conditions**. Columns with the same letter are not significantly different at *P* = 0.05.

The impact on cassava yields of the native AMF in single and dual inoculation was assessed in comparison to the commercial inoculant MykePro and the standard chemical fertilizer application (Figure 3). The results showed that the chemical fertilizer NPK significantly improved cassava yield (11.38 t/ha) compared to non-inoculated control (8.21 t/ha). This represents a yield gain of 38.5%. Of the AMF treatments, only *A. colombiana* single inoculation and the dual inoculation significantly (*p* = 0.003) improved cassava yield (9.58 and 9.81 t/ha, respectively) compared to non-inoculated control (8.21 t/ha). This represents a yield gain of 19.4% for the dual inoculation and 16.6% for *A. colombiana*. *A. appendicula* and the commercial inoculant had no significant impact on cassava yield compared to the non-inoculated control.

## Discussion

This work aimed to select an abundant native AMF capable of improving cassava crop productivity via several mechanisms, namely improved plant growth, water stress tolerance and nematode resistance. This is an improvement on previous studies, which tended to focus on one aspect affecting cassava yield, without studying the possible interactions with nematodes and drought.

During this study, there was a difference in the way the two native AMF species impacted cassava plant growth in greenhouse conditions. It was shown that only *A. colombiana* significantly increased the plant growth parameters, such as foliar surface area, plant height and biomass (Table 3). It has been reported that several factors, such as environmental conditions and functional diversity, can affect nutrient exchange between the fungi and plant partners (Walder and van der Heijden, 2015). The experimental conditions used in this work might have been favorable to *A. colombiana*, which significantly improved P uptake compared to *A. appendicula*. Variable effects among endogenous single species due to the use of different culture media were also observed in other studies (Williams et al., 2012; Ortas and Ustuner, 2014). Also, the two native AMF species may differ in terms of regulation of genes involved in *P* uptake. Such observations were made when maize plants were individually inoculated with different AMF species (Tian et al., 2013). In our study, single inoculation with *A. appendicula* had no effect. Meanwhile dual inoculation with both species positively improved P uptake and cassava plant growth. Similar observations were made when citrus was treated with different AMF species using a dual inoculation approach (Ortas and Ustuner, 2014). It could mean that when used together as dual inoculants, the two native AMF species induce phosphate transporters in cassava plants, as reported for different AMF species used to inoculate maize plants (Tian et al., 2013).

The study on the interaction between the two native AMF and *Meloidogyene* spp. in greenhouse revealed that negative effects of the AMF against the nematode (reduction of egg and nematode densities) were clearly observed, whether or not AMF and nematodes were co- or post-inoculated (1 month later). Interestingly, the presence of the nematode exerted a negative effect on the AMF, by reducing mycorrhizal intensities and frequencies in the case of simultaneous inoculation. Both types of interactions between nematode and AMF have already been reported. These mutual negative effects occur when fungi and nematodes are competing for space and nutrients (Schouteden et al., 2015). For example, the fungus *Scutellospora heterogama* exerted a biocontrol effect on the sedentary endoparasitic nematode *Meloidogyne incognita* (reproduction was reduced) only when it was pre-inoculated whereas co-inoculation had no effect (Dos Anjos et al., 2010). Such observations have also been made for migratory endoparasitic nematodes. For example, it was shown that *Radopholus similis* and *Pratylencus coffeae* affected the frequency of *Funneliformis mosseae* colonization in banana, but not the intensity (Elsen et al., 2003a,b). In contrast, root colonization by *R. irregularis in vitro* banana plantlets was not affected either by *R. similis* (Koffi et al., 2013) or by *P. coffeae* in transformed carrot roots (Elsen et al., 2003c).

Overall, in the presence of these native AMF, cassava plants continued to grow even though nematodes were present. It appears that the mycorrhizal cassava plants were either resistant (e.g., suppression or reduction of the nematode reproduction) or tolerant (low or no suppression in cassava plant growth) to nematodes, as reported in other studies (Hussey and Roncadori, 1982; Affokpon et al., 2011). However, the mechanism of the bioprotection conferred to cassava plants by the native AMF against the root-knot nematode *Meloidogyne* spp. is not yet understood. It may be due to the production of phytochemical inhibitors of nematodes, as was observed elsewhere. Indeed, in this work it was observed that phenolic compounds were significantly increased in cassava plant roots when nematodes were post-inoculated. Previous work has shown production of phenolic compounds to be a plant defense mechanism against nematode attacks (Zhu and Yao, 2004; Xu et al., 2008). Elsewhere, accumulation of phenolic compounds has been observed in mycorrhizal *Impatiens balsamina*, an ornamental plant, in presence of *M. incognita* (Banuelos et al., 2014). Singh et al. (1990) concluded that the pre-inoculation of plants, coupled with biochemical changes are responsible for resistance to nematodes. In contrast, when the cassava plants were co-inoculated with the AMF and the nematodes, there was no significant increase in phenolic compounds. Obviously there may be another mechanism involved in the inhibition of *Meloidogyne* spp. activity. For example, there was an up regulation of mycorrhiza-induced plant defense genes against the ectoparasitic nematode *Xiphinema index* in grapevine plants pre-inoculated with *R. intraradices* (Hao et al., 2012).

Besides its capacity to promote cassava growth and enhance resistance and tolerance to the root-knot nematode, *A. colombiana* also conferred water stress tolerance to cassava plants under severe drought condition. This AMF species significantly improved cassava plant growth under water stress. It was observed that mycorrhizal frequencies decreased gradually during the period of drought for all treatments, compared to the 100% FC water regime. However, despite severe water stress, the mycorrhizal colonization frequencies of *A. colombiana* remained higher than the control and stable over time. This water stress tolerance could be the result of *A. colombiana* promoting specific plant stress resistance response during the drought period, as suggested by others (Augé, 2001). For example the presence of this AMF may enhance photosynthetic activity due to the high levels of chlorophyll a and total sugars in *A. colombiana* colonized cassava plants compared to non-mycorrhizal plants (Mathur and Vyas, 1995).

Overall, this study clearly showed the multiple functions of the native AMF species *A. colombiana*. Importantly, *A. colombiana* was dominant in all three study areas and was persistently found and easily produced in trap culture. Abundance and persistence of AMF species are very important for efficient AMF species selection to ensure potential inocula are not lost during trap culture propagation (Trejo-Aguilar et al., 2013). This is essential, as the most widespread method for inoculum propagation is the use of trap plants (Berruti et al., 2016).

Under field conditions, *A. colombiana* showed a good potential for improving cassava productivity. The dual inoculation using the two native AMF species also increased cassava yield under field conditions. This opens up the possibility of using single and dual inoculation of these two native AMF species to improve cassava productivity in the field. During this study the native inoculants performed better than the commercial inoculant. Indeed, the origin and the composition of AMF are very important factors to take into account for inoculum development (Berruti et al., 2016). It has been shown that native AMF have higher efficiency in terms of plant protection against nematode (Affokpon et al., 2011) and stress tolerance (Ruiz-Lozano and Azcón, 2000) than commercial inoculants generally used in the field. Commercial inoculants are generally comprised of AMF species that can be considered as exotic species in tropical and subtropical regions (Oliveira et al., 2005; Schreiner, 2007). One main drawback in the use of commercial inoculants is the fact that the species used might not survive the competition with local AMF communities. Rodriguez and Sanders (2015), who discussed this issue, recommended research to understand local communities through metagenomics and genetic studies. The use of native inoculants comprised of native AMF like *A. colombiana* is highly recommended as an alternative to exotic species (Oliveira et al., 2005). As a persistent and abundant generalist, *A. colombiana* may have been a good competitor under field conditions, as in the greenhouse. Moreover, since commercial inoculants can be either ineffective (Faye et al., 2013) or badly formulated (Corkidi et al., 2004), the use of *A. colombiana* is more likely to be affordable and effective for cassava farmers in tropical and subtropical regions.

In conclusion, this study clearly points out the potential of *A. colombiana* as a native AM fungus suitable for inoculating cassava. The process developed in this study to select the multipurpose (plant growth improvement, water stress tolerance and nematode resistance) AMF species *A. colombiana* for cassava could be applied to efficiently select effective AMF inocula for other crops.

## Author contributions

This work is done is the scope of a project in the Laboratoire de Biotechnologie Végétale et Microbienne under the supervision of ZA. SJ designed and run all the experiments as a Ph.D. student. KC helped in designing the PCR amplification protocols and sequence analyses. VR was a cosupervisor of this work as a collaborator on this project. ZA is the coordinator of the project and SJ supervisor.

## Funding

We are grateful to the West African Agricultural Productivity Program (WAAPP) for funding the Project IVO-RHIZE (Projects 047/PPAAO/2012 and 028/CS/PPAAO/2015), within which this study was conducted. We are also grateful to this program for funding SJ scholarship and the Agence Universitaire de la Francophonie (AUF) for awarding him a biotechnology mobility program.

### Conflict of interest statement

The authors declare that the research was conducted in the absence of any commercial or financial relationships that could be construed as a potential conflict of interest.
